# Genistein Ameliorates Scopolamine-Induced Amnesia in Mice Through the Regulation of the Cholinergic Neurotransmission, Antioxidant System and the ERK/CREB/BDNF Signaling

**DOI:** 10.3389/fphar.2018.01153

**Published:** 2018-10-12

**Authors:** Cong Lu, Yan Wang, Teng Xu, Qi Li, Donghui Wang, Lijing Zhang, Bei Fan, Fengzhong Wang, Xinmin Liu

**Affiliations:** ^1^Institute of Food Science and Technology, Chinese Academy of Agricultural Sciences, Beijing, China; ^2^Research Center for Pharmacology and Toxicology, Institute of Medicinal Plant Development, Chinese Academy of Medical Sciences and Peking Union Medical College, Beijing, China

**Keywords:** genistein, scopolamine, memory deficits, cholinergic neurotransmission, antioxidant system, the ERK/CREB/BDNF signaling

## Abstract

Genistein (GE) was reported to exert a wide spectrum of biological activities, including antioxidant, anti-inflammatory, anti-mutagenic, anticancer, and cardio-protective effects. In addition, both clinical and preclinical studies have recently suggested GE a potential neuroprotective and memory-enhancing drug against neurodegenerative diseases. The animal model of scopolamine (Scop)-induced amnesia is widely used to study underlying mechanisms and treatment of cognitive impairment in neurodegenerative diseases. However, there is no report about the effects of GE on Scop-induced amnesia in mice. Therefore, the present study was carried out to investigate the beneficial effects and potential mechanism of GE against Scop-induced deficits in mice. The mice were orally pretreated with either GE (10, 20, and 40 mg/kg) or donepezil (1.60 mg/kg) for 14 days. After the pretreatment, the open field test was conducted to assess the effect of GE on the locomotor activity of mice. Thereafter, mice were daily injected with Scop (0.75 mg/kg) intraperitoneally to induce memory deficits and subjected to the cognitive behavioral tests including the Object Location Recognition (OLR) experiment and Morris Water Maze (MWM) task. After the behavioral tests, biochemical parameter assay and western blot analysis were used to examine the underlying mechanisms of its action. The results showed that GE administration significantly improved the cognitive performance of Scop-treated mice in OLR and Morris water maze tests, exerting the memory-enhancing effects. Additionally, GE remarkably promoted the cholinergic neurotransmission and protected against the oxidative stress damage in the hippocampus of Scop-treated mice, as indicated by decreasing AChE activity, elevating ChAT activity and Ach level, increasing SOD activity, lowering the level of MDA and increasing GSH content. Furthermore, GE was found to significantly upregulate the expression levels of p-ERK, p-CREB and BDNF proteins in the hippocampus of Scop-treated mice. Taken together, these results for the first time found that GE exerts cognitive-improving effects in Scop-induced amnesia and suggested it may be a potential candidate compound for the treatment of some neurodegenerative diseases such as Alzheimer’s Disease (AD).

## Introduction

Genistein, chemically known as 4′,5,7-trihydroxyisoflavone, is a non-steroidal polyphenol and is the most widely studied soy isoflavone so far (Mirahmadia et al., 2018). It has been demonstrated that GE possesses many physiological and pharmacological properties, ranging from antioxidant, anti-mutagenic and anticancer activities to cardio-protective and immunomodulatory capacities (Jeon et al., 2014; Ekambaram et al., 2016; Kang et al., 2016; Russo et al., 2016). In recent years, a growing number of both clinical and preclinical studies have suggested GE a potential neuroprotective and memory-enhancing drug against neurodegenerative diseases (Rahman et al., 2012; Du et al., 2018; Wu et al., 2018). It was reported that GE has exhibited the beneficial role against menopause-associated cognitive deficits and neurodegeneration through diminishing oxidative stress (Xu et al., 2007) and high consumption of GE in the diet has health-promoting benefits like memory improvement in volunteers of both genders (File et al., 2001). *In vivo* studies, accumulated evidence has showed that GE is capable to ameliorate several cognitive impairments animal models, such as OVX rats (Huang and Zhang, 2010; Khodamoradi et al., 2018), amyloid β(1–40) rat model of AD (Bagheri et al., 2011; Ding et al., 2013), LPS-induced memory deficits in rat and mice (Lee et al., 2017; Mirahmadia et al., 2018), Diabetes Associated Cognitive Decline (DACD) in mice (Rajput and Sarkar, 2017), 3-Nitropropionic acid (3-NPA) induced rat model of HD (Menze et al., 2015) and 6-hydroxydopamine hemi-Parkinsonian rat model (Baluchnejadmojarad et al., 2009). Furthermore, *in vitro* studies, GE exerted neuroprotective effects against amyloid β induced toxicity in SH-SY5Y human neuroblastoma cells (Bang et al., 2004; Park et al., 2016) and it was recently found that GE could balance the production and destruction of AChE by the activation of G protein-coupled receptor 30 in PC12 cell (Liu et al., 2018). Previous studies found that the neuroprotective effects of GE mainly involved several mechanisms such as resisting the oxidative stress damage, alleviating neuroinflammation, reducing Aβ deposition, activating the neurotrophins and antiapoptotic factors (Yang et al., 2014). Besides, as a lipid-soluble flavonoid compound, GE has the effective BBB penetration properties (Mozolewski et al., 2017). All these studies together suggest that GE may be a potential agent for the treatment of the neurodegenerative disease and it is noteworthy to further explore the neuroprotective effects of GE.

Scopolamine (Scop) is a non-selective post-synaptic muscarinic receptor blocker and can cause cognitive impairments in rodents and humans via decreasing the effectiveness of Ach in the CNS in animals and humans (Klinkenberg and Blokland, 2010; Kim et al., 2013). Scopolamine can induce the significant deficits in cognitive performance on behavioral tests, which makes it a valid pharmacological model for inducing cognitive (particularly mnemonic) deficits (Haider et al., 2016). However, there has been no research on the effect of GE against the Scop -induced amnesia in mice. Therefore, in the present study, to evaluate this effect, the mice were injected intraperitoneally with Scop to induce memory deficits. Behavioral studies were conducted to examine the learning and memory ability by OLR test and Morris Water Maze (MWM) task, while biochemical assays were used to investigate possible molecular mechanisms related.

## Materials and Methods

### Drugs and Reagents

Genistein (GE) was purchased from Shenggong Biological Engineering Co., Ltd. (D516BA0009, Shanghai, China) and the purity of GE exceeded 98.0% analyzed by HPLC. Carboxmethylcellulose (CMC-Na) was also obtained from Shenggong Biological Engineering Co., Ltd. (DA17BA0020, Shanghai, China). Scop was purchased from Sigma Aldrich Chemical Co., Ltd. (4A96820, St. Louis, MO, United States). Donepezil hydrochloride (Aricept) (DNP) was obtained from Eisai (111214A, Ibaraki, Japan). AChE (A024), ChAT (A079-1), Ach (A105-1), SOD (A001-3), MDA (A003-1), and GSH (A006-1) commercial kits were obtained from Jiancheng Biological Institute (Nanjing, Jiangsu, China).

### Animals

Sixty male Institute of Cancer Research mice (20–22 g) were purchased from Vital River Co., Ltd. (Qualified No.: SCXK 2016-0006, Beijing, China). All animals have free access to regular chow and water and were kept under a controlled 12 h light/dark (lights on at 8 AM) cycle at (23 ± 2)°C condition with the relative humidity at 50% ± 10%. All experimental procedures were performed under the approval and supervision of the Academy of Experimental Animal Center of the Institute of Medicinal Plant Development (No. 2017116-01) and in accordance with the National Institute of Health Guide for the Care and Use of Laboratory Animals. And all efforts were made to minimize the suffering of the animals.

### Drug Administration and Behavioral Assays

**FIGURE 1:**
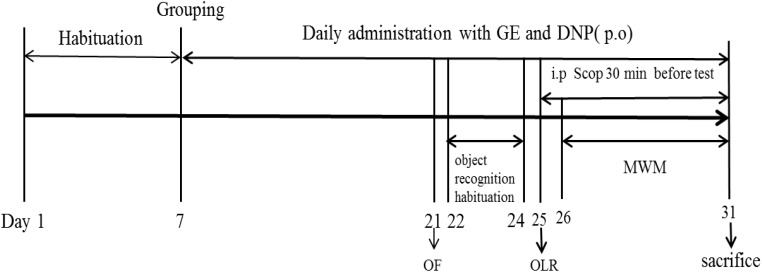
Experimental design procedure. After 7 days of the acclimatization period, the animals were randomly assigned to six groups: Control group; Scopolamine model group (Scop, 0.75 mg/kg); Scop + GE (L-10 mg/kg; M-20 mg/kg; H-40 mg/kg) groups and Scop + donepezil group (DNP, 1.60 mg/kg). Then, mice received oral pretreatment with GE and DNP for 14 days before inducing amnesia by Scop. After pretreatment, the open field (OF) test was firstly used to evaluate the effect of GE on the locomotor activity without Scop administration. Thereafter, cognitive deficits were induced by intraperitoneal administration of Scop (0.75 mg/kg) once daily and object location recognition (OLR) experiment and Morris water maze (MWM) task were examined. The Scop was injected intraperitoneally (i.p) to mice for 7 consecutive days, while GE and DNP were administrated orally for 24 consecutive days. Following the behavioral tests, all animals were sacrificed and their hippocampus were isolated for further biochemical analysis.

After 7 days of the acclimatization period, the animals were randomly assigned to six groups: Control group; Scop group (0.75 mg/kg); GE (L-10 mg/kg; M-20 mg/kg; H-40 mg/kg) groups and DNP group (1.60 mg/kg) with ten mice in each group. Then, the GE or DNP groups were pretreated with GE (10, 20, and 40 mg/kg; p.o.) or DNP (1.60 mg/kg; p.o) daily for 14 consecutive days. And the control and Scop groups received oral administration of the vehicle (0.5% CMC-Na). The dosage of GE used for this study was selected based on the previous report (Lee et al., 2017). Doses of Scop and DNP were determined according to our previous study (Lu et al., 2018). After pretreatment, the OF test was first used to evaluate the effect of GE on the locomotor activity without Scop administration. Thereafter, cognitive deficits were induced by intraperitoneal administration of Scop (0.75 mg/kg; i.p) once daily. The learning and memory was evaluated by the OLR experiment and the MWM task. The Scop was injected intraperitoneally (i.p) to mice for 7 consecutive days, while GE and DNP were administrated orally for 24 consecutive days. Following the behavioral tests, all animals were sacrificed and their hippocampus were isolated for further biochemical analysis. The experiment procedure was illustrated as shown in **Figure 1**.

### Open Field Test

The OF test was conducted to measure the locomotor activity of mice according to the method described previously (Dang et al., 2009). Briefly, it happened in an OF chamber which is a 30 cm × 28 cm × 35 cm open area with a 120 Lux light source in standard room lighting conditions and a video camera fixed at the top of each tank. The drug treatments were treated with GE or DNP (10, 20, 40, or 1.60 mg/kg, p.o.) 1 h before the test. The control and Scop groups received the vehicle and the experimenter for this test was blind to the treatment conditions of the tested mice. In the trial, each mouse was placed into the center of the OF chamber and allowed to explore freely for 3 min. Then a video tracking software system (developed by the China Astronaut Center and the Institute of Medicinal Plant Development, Chinese Academy of Medical Sciences, Beijing, China) automatically recorded the total distances and average speeding in the following 10 min as the indexes of locomotor activity. The floor of the chamber was cleaned with 70% ethanol after each trial.

### Object Location Recognition Test

The OLR test was used to evaluate the short-term, spatial object recognition memory (Ennaceur and Delacour, 1988) and the procedure was carried out as described previously (Lu et al., 2018). The apparatus consisted of a rectangular box (40 cm × 50 cm × 50 cm) which was made of a black polyester plastic material, camera mounted on the top of the chamber to record animals’ exploratory behavior. The objects (A1 and A2) used in this study were two small, washable plastic material bottles which were different in color but identical in shape and size (approximately 3 cm in diameter and 5height) and heavy enough to avoid overturned. The procedure was divided into three stages: habituation, familiarization, and test phases. In the habituation phase, mice were exposed to explore the box (with no objects) for 10 min to acclimatize to the apparatus and reduce the animals’ fear of a new environment. The 10 min-habituation session was repeated for three consecutive days. On the fourth day, the experimental trials began, which consisted of a familiarization phase and a test phase with a 30 min interval between each trial. During the interval, the mouse was placed in the holding cage, which remained inside the testing room. During the familiarization phase, mice were placed in arena, which contained two identical objects and mice were allowed to freely explore the box (5 min per trial). After a 30 min delay period, a test trial was conducted; mice were returned to arena in which one of the original objects had changed location (“novel”) and the other object remained in the original position (“familiar”). Objects and their placement into the arena were varied counterbalanced between groups to avoid positional biases. To control for possible odor cues, the objects and the floor of the arena were cleaned with 70% ethanol at the end of each trial to eliminate possible scent/trail markers. Exploratory behavior was considered only when the mice were sniffing or touching the object with the nose. In each trial, the duration of contact with each object was recorded using a stopwatch. Animal exploratory behavior was recorded using a video camera, and all behaviors were scored from the video to ensure accuracy. The exploration time for A1and A2 object and the total exploration time (Te) were recorded in the familiarization phase. In the test phase, recognition memory was evaluated by the DI calculated as time spent exploring the “novel” object compared with the “familiar” object relative to the total time spent exploring all objects, according to the formula: DI = (TN-TF)/(TN + TF) [TN = time spent exploring the “novel” object; TF = time spent exploring the “familiar” object] (Khajevand-Khazaei et al., 2018).

### Morris Water Maze Task

Morris water maze task was performed subsequently to assess the long-term, spatial reference memory in mice and the protocol of MWM was conducted according to a published method (Wang et al., 2010). The test device consisted of a circular, black pool (1.0 m in diameter and 0.38 m in height) and filled with black ink at the temperature of (23 ± 2) °C to a depth of 25 cm. The pool was divided into four identical quadrants (marked NE, SE, SW, and NW) with a submerged (1.0 cm beneath water surface) platform (6.0 cm in diameter and 15 cm in height) positioned in one of the four maze quadrants, labeled as the target quadrant. A video camera was used to record the performance of animals in the pool. The acquisition test consisted of five consecutive daily trials in which mice were placed (facing the wall) 3 times into each of four quadrants in turn (except for target quadrant) and must learn to locate the hidden platform beneath in water. Before each trial, mice were placed on the hidden platform for 10 s to be trained to remember the platform. Then they were gently released from one of four quadrants randomly and allowed to find the platform in 90 s. Learning and memory was measured by the escape latency. If mice did not locate the hidden platform within 90 s, the mice were gently guided to the platform, allowed to stay on the platform for 10 s, and the escape latency was recorded for 90 s. On the sixth day, after the completion of the acquisition trial, the probe test was conducted to assess spatial reference memory of the animals, in which the platform was removed. The mice were placed into the pool side opposite to the target quadrant and they were probed in a 90 s “retention” trial. The crossing numbers of the target platform quadrant was recorded to reflect the retention memory for the platform location. Mouse activity in the pool was recorded by a digital video camera connected to a computer-controlled software system (developed by the China Astronaut Center and the Institute of Medicinal Plant Development, Chinese Academy of Medical Sciences, Beijing, China).

### Biochemical Assay

Following the behavioral tests, the mice were sacrificed and the brains were quickly removed. Then the isolated hippocampal tissues from both hemispheres were promptly excised, and immediately snap-frozen and stored at –80°C for further analysis. For the biochemical detection, the hippocampus was weighed and homogenized in 9 vol (1:9, w/v) ice-cold 0.9% normal saline. After centrifugation at 3,000 rpm for 10 min at 4°C, the supernatant was collected and further diluted with appropriate buffer solution for the determination of the relevant biochemical parameters. AChE, ChAT, SOD activities and contents of Ach, MDA, and GSH in the hippocampus were measured using commercially available kits (Nanjing Jiancheng Bioengineering Institute, Jiangsu, China), respectively, according to the manufacturer’s instructions.

### Western Blot Analysis

The Western blot method was performed as previously described (Lu et al., 2018). The hippocampal tissues were homogenized on ice in CellLytic MT mammalian tissue lysis reagent (C3248, Sigma-Aldrich, St. Louis, MO, United States) containing protease and phosphatase inhibitor cocktails (P3850, Sigma-Aldrich, St. Louis, MO, United States). The homogenate was centrifuged at 10,000 ×*g* for 15 min at 4°C and the protein concentration was further determined by using an enhanced BCA protein assay kit (Thermo scientific, Waltham, MA, United States). Protein samples (30 μg/sample) were electrophoresed on a 10% Sodium Dodecyl Sulfate PolyAcrylamide Gel Electrophoresis (SDS–PAGE) and transferred onto PVDF membranes (Millipore, Burlington, MA, United States). The membranes were blocked for 1 h using 5% non-fat dry milk in Tris-Buffered Saline (TBS) containing 0.5% Tween-20 (TBST), then probed with specific primary antibodies against phosphor ERK1/2, ERK 1/2, phosphor CREB, and CREB, BDNF (1: 500), and β-actin (Cell Signaling Technology, Danvers, MA, United States) overnight at 4°C. After thoroughly washed with PBST (PBS with 0.1% Tween 20), the membrane was incubated with HRP-conjugated secondary antibodies at room temperature for another 1 h. The protein bands were visualized with ECL prime kit (GE Healthcare, NA, United Kingdom).

### Statistical Analysis

All data were analyzed using the SPSS 19.0 software package (Chicago, IL, United States) and represented as means ± SEM. The escape latency in the acquisition trials of MWM was analyzed using repeated-measure two-way ANOVA and Paired *T*-test was conducted to compare the exploration time for the object A1 and the object A2 in the familiarization phase of OLR. The total distance and average speeding of the OF test, the DI of the OLR task, the crossing number of MWM test, the data of biochemical assays and WB were Analyzed by One-Way Analysis of Variation (ANOVA) followed by LSD test for multiple comparisons and performed using GraphPad Prism software 5.0 (GraphPad Prism Software, CA, United States). For all statistical tests, *P* < 0.05 was considered significant.

## Results

### Ge Did Not affect the on Locomotor Activities of Mice

**FIGURE 2:**
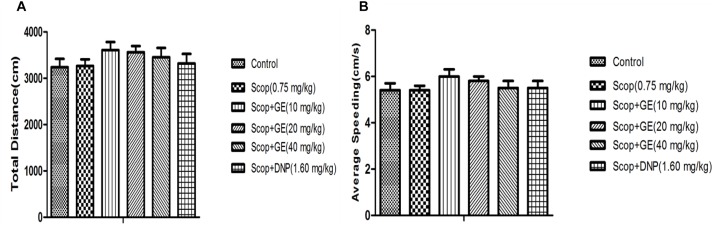
Effect of GE on locomotor activities of mice. **(A)** Total distance, **(B)** Average speeding. Data are represented as the mean value ± SEM. *n* = 10 in each group.

As shown in **Figure 2**, there were no significant changes in total distance and average speeding among all groups, suggesting that pretreatment with GE did not affect the locomotor activity of mice. The results of the OF test could eliminate its interference in cognitive function of mice.

### GE Improved the Short-Term, Spatial Memory of Scop-Treated Mice in OLR Test

**FIGURE 3:**
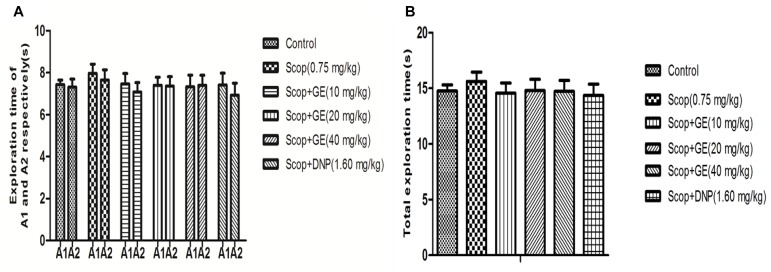
Effect of GE on the ability of exploration in the familiarization phase of OLR task. **(A)** Exploration time of A1 and A2 respectively of mice, **(B)** Total exploration time. Data are represented as the mean value ± SEM. *n* = 10 in each group.

**FIGURE 4:**
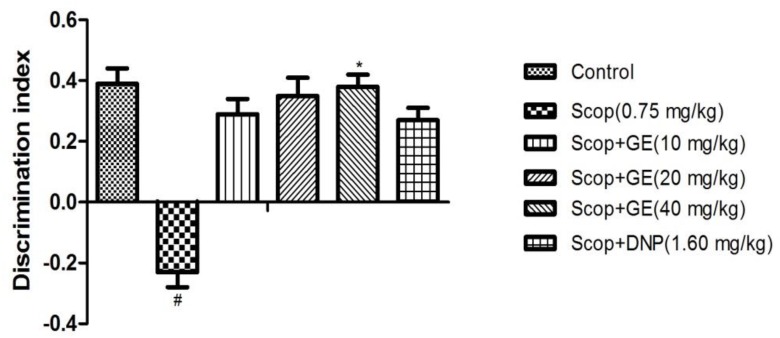
Effect of GE on short-term, spatial memory in the test phase of OLR task. Data are represented as the mean value ± SEM, *n* = 10 in each group. Significant differences ^#^*P* < 0.05 compared to the control group. ^∗^*P* < 0.05 compared with the Scop group.

The effects of GE on the OLR memory were examined (**Figures 3**, **4**). In the familiarization phase, no significant differences were observed for the exploration time of the object A1 and the object A2 comparing between groups (**Figure 3A**), thus there were no obvious changes in the total exploration time among all groups (**Figure 3B**). The results guaranteed that there were no alterations in the animals’ exploration ability and location preference.

In the test phase, as shown in **Figure 4**, when comparing among groups, differences were found for the DI [*F*_(5,56)_ = 1.673, *P* < 0.05]. Multiple *post hoc* comparisons analysis showed that Scop treatment significantly (*P* < 0.05) decreased the DI compared to the control group, indicating the memory impairment successfully induced by Scop. However, treatment with GE (40 mg/kg) significantly reversed it by elevating the DI compared with the Scop group and its action almost equalized the performance of the control group.

### GE Improved the Long-Term, Spatial Memory of Scop-Treated Mice in MWM Task

**FIGURE 5:**
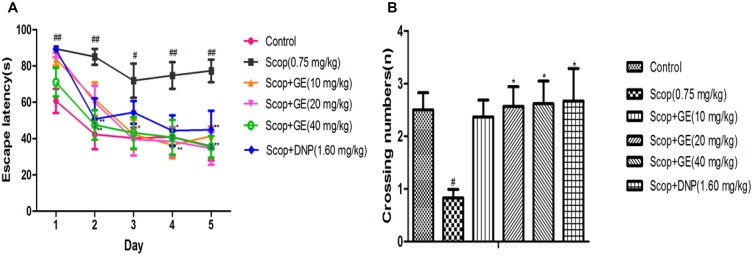
Effect of GE on the long-term, spatial reference memory in MWM task. **(A)** Escape latency in the acquisition phase, **(B)** Virtual-platform crossing numbers in the probe trial. Data are represented as the mean value ± SEM, *n* = 10 in each group. Significant differences ^#^*P* < 0.05, ^##^*P* < 0.01 compared to the control group; ^∗^*P* < 0.05, ^∗∗^*P* < 0.01 compared with the Scop group.

Effects of GE on spatial reference memory were assessed by MWM task. In the acquisition phase, as shown in **Figure 5A**, Scop-treated mice had longer escape latency than the control mice from the first to the fifth day [*F*_(5,56)_ = 3.086, *P* < 0.01; *F*_(5,56)_ = 2.743, *P* < 0.01; *F*_(5,56)_ = 2.629, *P* < 0.05; *F*_(5,56)_ = 3.167, *P* < 0.01; *F*_(5,56)_ = 2.948, *P* < 0.01; respectively], while GE (10, 20, and 40 mg/kg) administrations could significantly shorten this escape latency prolongation from the third to fifth day; the third to fifth day and the second to fifth day, respectively (*P* < 0.05, *P* < 0.05, *P* < 0.01). In addition, DNP (1.60 mg/kg) obviously (*P* < 0.05) reduced the escape latency at the second, fourth and fifth day compared with Scop group.

In the probe test, the crossing number in Scop group was significantly [*F*_(5,56)_ = 2.573, *P* < 0.05] decreased compared to the control group, however, treatment with GE (20 and 40 mg/kg) and DNP (1.60 mg/kg) markedly increased the crossing numbers of Scop-treated mice (**Figure 5B**).

### GE Decreased AChE Activity, Increased ChAT Activity and Elevated Ach Level in the Hippocampus of Scop-Treated Mice

**FIGURE 6:**
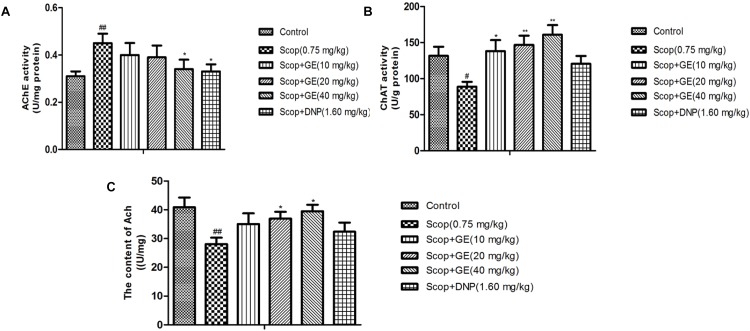
Effect of GE on **(A)** AChE activity, **(B)** ChAT activity and **(C)** Ach level in the hippocampus of Scop-treated mice. Data are represented as the means ± SEM, *n* = 10 in each group. Significant differences ^#^*P* < 0.05, ^##^*P* < 0.01 compared to the control group; ^∗^*P* < 0.05, ^∗∗^*P* < 0.01 compared with the Scop group.

As shown in **Figure 6A**, Scop treatment exhibited a significant increase [*F*_(5,56)_ = 2.318, *P* < 0.01] in the AChE activity in the hippocampus compared to the control group. Significant reductions of the AChE activity were observed in GE (40 mg/kg) and DNP (1.60 mg/kg) groups compared with Scop group (*P* < 0.05). The ChAT activity and Ach level in the hippocampus of Scop-treated mice were significantly (*P* < 0.05) lower than those of control group mice. Treatment with different doses of GE significantly (*P* < 0.05) increased the ChAT activity and Ach level in the hippocampus of Scop-treated mice (**Figures 6B,6C**).

### GE Increased SOD Activity, Lowered MDA Level and Increased GSH Content in the Hippocampus of Scop-Treated Mice

**FIGURE 7:**
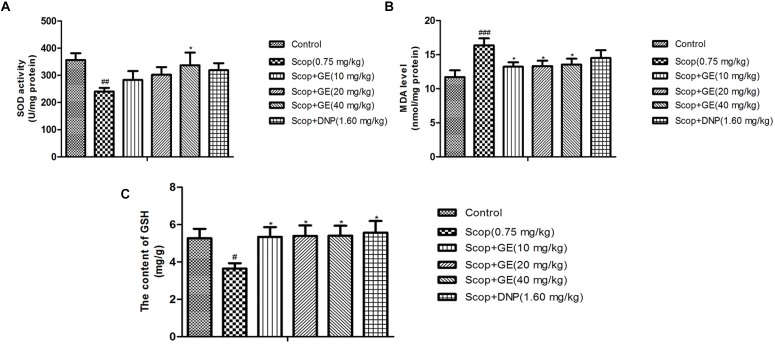
Effect of GE on **(A)** SOD activity, **(B)** MDA level and **(C)** GSH content in the hippocampus of Scop-treated mice. Data are represented as the means ± SEM, *n* = 10 in each group. Significant differences ^#^*P* < 0.05, ^##^*P* < 0.01, and ^###^*P* < 0.001 compared to the control group; ^∗^*P* < 0.05 compared with the Scop group.

As shown in **Figure 7**, Scop treatment induced oxidative stress damage indicated by the significant lower SOD activity, higher MDA level and decreased GSH content in the hippocampus [**Figure 7A**, *F*_(5,56)_ = 18.682, *P* < 0.01; **Figure 7B**, *F*_(5,56)_ = 9.393, *P* < 0.001; and **Figure 7C**, *F*_(5,56)_ = 3.576, *P* < 0.05]. Treatment with GE (40 mg/kg) obviously (*P* < 0.05) increased the SOD activity of Scop-treated mice. Additionally, with GE (10, 20 and 40 mg/kg) administrations, the increased MDA level and lowered GSH content induced by Scop treatment were ameliorated with significance (*P* < 0.05).

### GE Promoted the Expressions of p-EKR/ERK, p-CREB/CREB and BDNF Protein in the Hippocampus of Scop-Treated Mice

**FIGURE 8:**
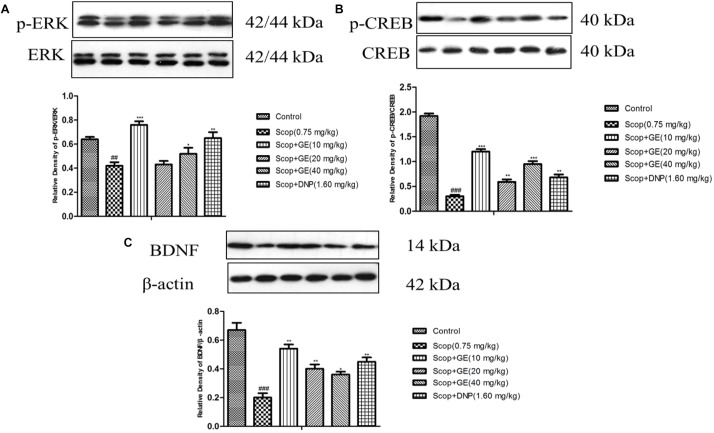
Effect of GE on the protein expressions of **(A)** p-ERK and ERK, **(B)** p-CREB and CREB, and **(C)** BDNF in the hippocampus of Scop-treated mice. Data are represented as the means ± SEM, *n* = 3 in each group. Significant differences ^##^*P* < 0.01, ^###^*P* < 0.001 compared to the control group; ^∗^*P* < 0.05, ^∗∗^*P* < 0.01, and ^∗∗∗^*P* < 0.001 compared with the Scop group.

Western blot analysis results showed that Scop treatment significantly decreased p-ERK/ERK [**Figure 8A**, *F*_(5,__56)_ = 2.018, *P* < 0.01], p-CREB/CREB [**Figure 8B**, *F*_(5,56)_ = 3.323, *P* < 0.001] and BDNF expression level [**Figure 8C**, *F*_(5,56)_ = 3.847, *P* < 0.001]. However, treatments with GE (10 and 40 mg/kg) and DNP (1.60 mg/kg) effectively (*P* < 0.001) recovered the Scop-suppressed ERK phosphorylation, CREB phosphorylation and BDNF expression levels in the hippocampus. In addition, GE (20 mg/kg) administration significantly (*P* < 0.01) upregulated the expression of p-CREB/CREB and BDNF proteins in the hippocampus compared to the Scop group (**Figures 8B,8C**).

## Discussion

Our present study provided the first evidence that GE has the neuroprotective effects on Scop -induced amnesia in mice. The results demonstrated that GE administration alleviated the cognitive deficits induced by Scop in both OLR and MWM tasks. And its action may be related to promote the cholinergic neurotransmission, enhance the antioxidant system and activate the ERK/CREB/BDNF signaling.

In the present study, two types of cognitive memory tasks (OLR and MWM) were utilized to evaluate the effects of GE on Scop-treated amnesia in mice. The OLR test based on the rodent spontaneity to explore a “novel” object was first carried out to assess the short-term, spatial recognition memory (Xu et al., 2016). Our current results demonstrated that Scop significantly decreased the DI in OLR test, which was consistent with the previous studies (Hooge and Deyn, 2001; Lu et al., 2018). For the first time, GE (40 mg/kg) treatment was found to robustly improve the impaired OLR memory in the Scop-treated mice, indicating the short-term spatial memory-enhancement effects. The MWM task was thereafter conducted to evaluate the long-term, spatial learning ability and reference memory (Zhao et al., 2016). The escape latency in the acquisition test could be used to reflect the spatial learning and memory ability, which means that mice must learn the accurate position of the hidden platform and develop suitable swimming approaches to reach it by distinguishing visual cues in the test room. The virtual-platform crossing numbers in the probe test is a key indicator assessed reference memory when the platform was absent. In agreement with the previous studies (Rajput and Sarkar, 2016; Vasileva et al., 2016), Scop administration markedly prolonged the escape latency in the acquisition phase and decreased the virtual-platform crossing numbers in the probe trial. However, the impaired memory ability induced by Scop was reversed by GE treatment. The long-term, spatial reference memory-improvements of GE administration were also observed in the previous study (Lee et al., 2014), which found that GE administration improved seizure-induced memory impairment and diabetes associated cognitive decline in the MWM task. Furthermore, the OF test was performed to rule out any confounding motor impairments that can influence outcomes in the cognitive behavioral tests (Parfitt et al., 2012). No significant differences in locomotor activity of mice were observed among groups, suggesting that treatment with GE had no effect on sensorimotor performance. These results of the behavioral studies confirmed that GE had the neuroprotective effects against Scop-induced memory impairments.

The central cholinergic neurotransmission plays an important role in cognitive functions, and drugs affecting it have been shown to change performance in tests of learning and memory (Brandon et al., 2004). As a neurotransmitter essential for learning and memory, Ach is synthesized by ChAT in cholinergic neurons and hydrolyzed by AChE after its release (Hut and Van der Zee, 2011). The expression and activation of AChE and ChAT regulate the dynamic concentration of Ach in the cholinergic synapses (Pachauri et al., 2012). Therefore, in order to elucidate the underlying mechanism of beneficial effect of GE in Scop-treated mice, AChE and ChAT activities as well as Ach level in the hippocampus were measured. Our present findings revealed that treatment with GE significantly reduced the AChE activity, increased ChAT activity and elevated Ach level in the hippocampus as compared to the Scop-treated mice. Similarly, it was recently reported that chronic GE treatment significantly improved cognitive performance in diabetic mice by affecting AChE activity (Rajput and Sarkar, 2017). The results suggested that inhibition of AChE activity and enhancement of ChAT activity by GE administration may have a protective role in Ach degradation and improved the cholinergic neurotransmission. In addition, Scop -induced amnesia was also related to the increased oxidative stress in the brain, especially in the hippocampus associated with spatial learning and memory (Kelleher et al., 2004). In fact, numerous studies have implicated that oxidative stress plays a key role in the pathogenesis of neurodegenerative disorders and the antioxidants from natural products are thought to be substances capable of protecting normal neuronal cells from the oxidative stress-induced death or damage that leads to cognitive decline (Bellingham, 2011; Lee et al., 2017). Genistein, an isoflavone found primarily in legumes (mainly soyabean), has been found to scavenge oxygen-derived free radicals and is able to activate antioxidant systems (Ganai and Husain, 2017). Hence, in our present study, the antioxidant action of GE was also measured. In agreement, our current results demonstrated that Scop significantly increased oxidative stress in the mouse hippocampus, as shown by decreased antioxidant enzyme SOD activity, increased an indicator of lipid peroxidation-MDA level and reduced non-enzymatic antioxidant GSH content. GE treatment significantly increased SOD activity, attenuated elevated MDA level and elevated GSH content in the Scop-treated mice, showing the antioxidant action. It was well consistent with the previous reports that GE had neuroprotective properties by reducing oxidative stress (Bagheri et al., 2011; Lee et al., 2014). Therefore, based on these results, GE may ameliorate the cognitive deficits caused by Scop via enhancement of the cholinergic neurotransmission and antioxidant action in mice.

The ERK is thought to play a critical role in synaptic plasticity, learning and memory (Yamashima, 2012). CREB, a nuclear transcription factor, is critical for hippocampus-dependent memory and activation of CREB by phosphorylation is necessary for its function (Seely et al., 2012). ERK pathway activation is coupled to CREB phosphorylation at Ser133 site and ERK-mediated CREB phosphorylation is required for the induction of LTP and long-term memory (Lu, 2003). The best known transcriptional target of CREB is BDNF which is known to emerge as an important synaptic modulator of synaptogenesis (Jiang et al., 2017). Thus, the ERK/CREB/BDNF signaling was suggested to be involved in the cognitive performance. According to the previous literature, GE was reported to exert the neuroprotective effects on the isoflurane-induced neurotoxicity by activating cAMP/CREB-BDNF-TrkB-PI3/Akt signaling (Samrat et al., 2015) and it significantly attenuated LPS-induced cognitive dysfunction in mice by increasing BDNF expression and CREB phosphorylation (Lee et al., 2017). In addition, several lines of evidence indicated that Scop -induced memory deficits were associated with significant inhibitions of the ERK, CREB phosphorylation, and BDNF expression levels in the hippocampus (Seely et al., 2012; Hafez et al., 2017). Therefore, in our present study, whether GE administration could affect the ERK/CREB/BDNF signaling in the Scop-treated mouse hippocampus was examined. Our findings in line with previous studies demonstrated that GE administration upregulated ERK, CREB phosphorylation and BDNF expression level in the Scop-treated mouse hippocampus. Taking into account that the functional role of these molecules in regulating learning and memory, the activation of the ERK/CREB/BDNF signaling could account for the neuroprotective effects of GE on Scop -induced memory deficits.

## Conclusion

In summary, GE could alleviate Scop -induced amnesia in mice through promoting the cholinergic neurotransmission, enhancing antioxidant system and activating the ERK/CREB/BDNF signaling. All these results suggested that GE may be a potential candidate compound for the treatment of some neurodegenerative diseases such as AD.

## Author Contributions

CL, FW, and XL participated in the experiment design. CL, TX, and QL conducted the experiments and performed the data analysis. CL, YW, DW, LZ, BF, and XL contributed to the writing and amendments of the manuscript.

## Conflict of Interest Statement

The authors declare that the research was conducted in the absence of any commercial or financial relationships that could be construed as a potential conflict of interest.

## Abbreviation

Ach: acetylcholine
AChE: acetylcholinesterase
AD: Alzheimer’s disease
ANOVA: analysis of variance
BBB: blood-brain barrier
BCA: bicinchoninic acid
BDNF: brain-derived neurotrophic factor
ChAT: choline acetyltransferase
CMC-Na: Carboxymethylcellulose
CNS: central nervous system
CREB: cyclic AMP response element binding protein
DNP: donepezil
DI: discrimination index
ERK: extracellular signal-regulated kinase
GE: genistein
GSH: glutathione
HD: huntington’s disease
HRP: horseradish peroxidase
SEM: standard error mean
LSD: least significant digit
LPS: lipopolysaccharide
LTP: long-term potentiation
MDA: malondialdehyde
MWM: morris water maze task
OF: open field
OLR: object location recognition
OVX: ovariectomized
PBS: phosphate buffered solution
PVDF: polyvinylidene difluoride
Scop: scopolamine
SDS–PAGE: sodium dodecyl sulfate polyacrylamide gel electrophoresis
SOD: superoxide dismutase
TBARS: thiobarbituric acid reactive substance
